# 3-Nitro­phenyl pyrimidin-2-yl ether

**DOI:** 10.1107/S1600536809007697

**Published:** 2009-03-06

**Authors:** Nasir Shah Bakhtiar, Zanariah Abdullah, Seik Weng Ng

**Affiliations:** a Department of Chemistry, University of Malaya, 50603 Kuala Lumpur, Malaysia

## Abstract

In the title compound, C_10_H_7_N_3_O_3_, the dihedral angle between the two aromatic rings is 87.5 (1) Å; their *ipso*-C atoms subtend an angle of 117.4 (1)° at the ether O atom.

## Related literature

For the structure of phenyl pyrimidin-2-yl ether, see: Shah Bakhtiar *et al.* (2009 ▶).
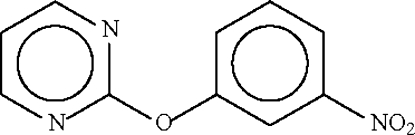


## Experimental

### 

#### Crystal data

C_10_H_7_N_3_O_3_
*M* *_r_* = 217.19
Orthorhombic, 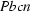

*a* = 18.1360 (3) Å
*b* = 7.3355 (1) Å
*c* = 14.5986 (3) Å
*V* = 1942.15 (6) Å^3^
*Z* = 8
Mo *K*α radiation
μ = 0.11 mm^−1^
*T* = 118 K
0.40 × 0.20 × 0.15 mm

#### Data collection

Bruker SMART APEX CCD diffractometer
Absorption correction: none
12785 measured reflections
2242 independent reflections
1890 reflections with *I* > 2σ(*I*)
*R*_int_ = 0.029

#### Refinement

*R*[*F* ^2^ > 2σ(*F* ^2^)] = 0.037
*wR*(*F* ^2^) = 0.102
*S* = 1.02
2242 reflections
145 parameters
H-atom parameters constrained
Δρ_max_ = 0.27 e Å^−3^
Δρ_min_ = −0.29 e Å^−3^

### 

Data collection: *APEX2* (Bruker, 2007 ▶); cell refinement: *SAINT* (Bruker, 2007 ▶); data reduction: *SAINT*; program(s) used to solve structure: *SHELXS97* (Sheldrick, 2008 ▶); program(s) used to refine structure: *SHELXL97* (Sheldrick, 2008 ▶); molecular graphics: *X-SEED* (Barbour, 2001 ▶); software used to prepare material for publication: *publCIF* (Westrip, 2009 ▶).

## Supplementary Material

Crystal structure: contains datablocks global, I. DOI: 10.1107/S1600536809007697/hb2922sup1.cif

Structure factors: contains datablocks I. DOI: 10.1107/S1600536809007697/hb2922Isup2.hkl

Additional supplementary materials:  crystallographic information; 3D view; checkCIF report

## Figures and Tables

**Fig. 1.**
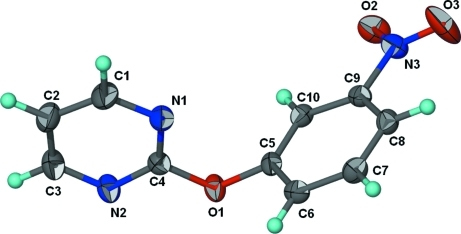
The molecular structure of (I) with displacement ellipsoids drawn at the 70% probability level. Hydrogen atoms are drawn as spheres of arbitrary radius.

## supplementary crystallographic information

### Experimental

3-Nitrophenol (2.78 g, 20 mmol) was mixed with sodium hydroxide (0.08 g, 20 mmol) in several drops of water. The water was then evaporated. The paste was heated with 2-chloropyrimidine (2.30 g, 20 mmol) at 423–433 K for 6 h. The product was dissolved in water and the solution extracted with chloroform. The chloroform phase was dried over sodium sulfate; the evaporation of the solvent gave well shaped very pale brown blocks of (I) 40% yield along with some unidentified brown material.

### Refinement

The H-atoms were placed in calculated positions (C—H 0.95 Å) and refined as riding with *U*(H) = 1.2*U*_eq_(C).

### Crystal data

**Table:**

C_10_H_7_N_3_O_3_	*F*(000) = 896
*M**_r_* = 217.19	*D*_x_ = 1.486 Mg m^−^^3^
Orthorhombic, *P**b**c**n*	Mo *K*α radiation, λ = 0.71073 Å
Hall symbol: -P 2n 2ab	Cell parameters from 4130 reflections
*a* = 18.1360 (3) Å	θ = 2.8–28.2°
*b* = 7.3355 (1) Å	µ = 0.11 mm^−^^1^
*c* = 14.5986 (3) Å	*T* = 118 K
*V* = 1942.15 (6) Å^3^	Block, faint brown
*Z* = 8	0.40 × 0.20 × 0.15 mm

### Data collection

**Table:**

Bruker SMART APEX CCD diffractometer	1890 reflections with *I* > 2σ(*I*)
Radiation source: fine-focus sealed tube	*R*_int_ = 0.029
graphite	θ_max_ = 27.5°, θ_min_ = 2.3°
ω scans	*h* = −23→23
12785 measured reflections	*k* = −9→9
2242 independent reflections	*l* = −18→18

### Refinement

**Table:**

Refinement on *F*^2^	Primary atom site location: structure-invariant direct methods
Least-squares matrix: full	Secondary atom site location: difference Fourier map
*R*[*F*^2^ > 2σ(*F*^2^)] = 0.037	Hydrogen site location: inferred from neighbouring sites
*wR*(*F*^2^) = 0.102	H-atom parameters constrained
*S* = 1.02	*w* = 1/[σ^2^(*F*_o_^2^) + (0.0556*P*)^2^ + 0.7122*P*] where *P* = (*F*_o_^2^ + 2*F*_c_^2^)/3
2242 reflections	(Δ/σ)_max_ = 0.001
145 parameters	Δρ_max_ = 0.27 e Å^−^^3^
0 restraints	Δρ_min_ = −0.29 e Å^−^^3^

### Fractional atomic coordinates and isotropic or equivalent isotropic displacement parameters (Å^2^)

**Table:**

	*x*	*y*	*z*	*U*_iso_*/*U*_eq_	
O1	0.46423 (5)	0.52259 (12)	0.36904 (6)	0.0222 (2)	
O2	0.34305 (5)	0.73218 (14)	0.08352 (6)	0.0291 (2)	
O3	0.22956 (6)	0.7828 (2)	0.12079 (7)	0.0467 (3)	
N1	0.42087 (6)	0.22878 (14)	0.35641 (8)	0.0228 (2)	
N2	0.54579 (6)	0.29874 (15)	0.39501 (8)	0.0234 (3)	
N3	0.29278 (6)	0.73707 (16)	0.13953 (7)	0.0238 (3)	
C1	0.43734 (8)	0.05109 (18)	0.36142 (10)	0.0280 (3)	
H1	0.3994	−0.0355	0.3502	0.034*	
C2	0.50707 (8)	−0.01134 (18)	0.38219 (10)	0.0284 (3)	
H2	0.5182	−0.1378	0.3850	0.034*	
C3	0.56002 (7)	0.12058 (19)	0.39866 (10)	0.0267 (3)	
H3	0.6087	0.0824	0.4132	0.032*	
C4	0.47660 (7)	0.33980 (16)	0.37377 (8)	0.0180 (3)	
C5	0.39288 (6)	0.58007 (15)	0.34572 (8)	0.0179 (3)	
C6	0.34150 (7)	0.60883 (16)	0.41443 (8)	0.0205 (3)	
H6	0.3532	0.5820	0.4764	0.025*	
C7	0.27269 (7)	0.67742 (17)	0.39132 (9)	0.0218 (3)	
H7	0.2369	0.6965	0.4378	0.026*	
C8	0.25564 (7)	0.71852 (17)	0.30078 (8)	0.0202 (3)	
H8	0.2086	0.7655	0.2845	0.024*	
C9	0.30919 (7)	0.68896 (16)	0.23527 (8)	0.0181 (3)	
C10	0.37846 (6)	0.61910 (15)	0.25487 (8)	0.0180 (2)	
H10	0.4141	0.5991	0.2083	0.022*	

### Atomic displacement parameters (Å^2^)

**Table:**

	*U*^11^	*U*^22^	*U*^33^	*U*^12^	*U*^13^	*U*^23^
O1	0.0150 (4)	0.0171 (4)	0.0344 (5)	−0.0013 (3)	−0.0053 (4)	0.0031 (4)
O2	0.0272 (5)	0.0406 (6)	0.0196 (5)	−0.0023 (4)	0.0039 (4)	0.0012 (4)
O3	0.0262 (6)	0.0873 (10)	0.0266 (6)	0.0152 (6)	−0.0062 (4)	0.0083 (6)
N1	0.0197 (5)	0.0192 (5)	0.0295 (6)	−0.0015 (4)	−0.0035 (4)	−0.0006 (4)
N2	0.0175 (5)	0.0233 (6)	0.0294 (6)	0.0008 (4)	−0.0015 (4)	0.0036 (4)
N3	0.0215 (5)	0.0300 (6)	0.0199 (5)	0.0000 (4)	−0.0019 (4)	0.0000 (4)
C1	0.0278 (7)	0.0187 (6)	0.0375 (8)	−0.0030 (5)	−0.0046 (6)	−0.0024 (5)
C2	0.0300 (7)	0.0199 (6)	0.0352 (7)	0.0038 (5)	−0.0011 (6)	−0.0005 (5)
C3	0.0209 (6)	0.0265 (7)	0.0326 (7)	0.0050 (5)	−0.0013 (5)	0.0033 (5)
C4	0.0183 (6)	0.0186 (6)	0.0173 (6)	−0.0010 (4)	0.0003 (4)	0.0019 (4)
C5	0.0150 (5)	0.0133 (5)	0.0255 (6)	−0.0016 (4)	−0.0025 (5)	0.0005 (4)
C6	0.0237 (6)	0.0193 (6)	0.0186 (6)	−0.0031 (5)	−0.0010 (5)	0.0015 (4)
C7	0.0215 (6)	0.0224 (6)	0.0214 (6)	0.0007 (5)	0.0046 (5)	−0.0013 (5)
C8	0.0154 (5)	0.0213 (6)	0.0240 (6)	0.0022 (4)	0.0007 (5)	−0.0010 (5)
C9	0.0185 (6)	0.0182 (5)	0.0175 (6)	−0.0013 (4)	−0.0012 (4)	−0.0005 (4)
C10	0.0152 (5)	0.0173 (5)	0.0214 (6)	−0.0010 (4)	0.0023 (4)	−0.0021 (4)

### Geometric parameters (Å, °)

**Table:**

O1—C4	1.3613 (15)	C2—H2	0.9500
O1—C5	1.4029 (14)	C3—H3	0.9500
O2—N3	1.2252 (14)	C5—C10	1.3819 (17)
O3—N3	1.2256 (15)	C5—C6	1.3852 (17)
N1—C4	1.3225 (16)	C6—C7	1.3872 (18)
N1—C1	1.3392 (17)	C6—H6	0.9500
N2—C4	1.3273 (16)	C7—C8	1.3907 (18)
N2—C3	1.3332 (17)	C7—H7	0.9500
N3—C9	1.4720 (16)	C8—C9	1.3801 (17)
C1—C2	1.379 (2)	C8—H8	0.9500
C1—H1	0.9500	C9—C10	1.3867 (17)
C2—C3	1.384 (2)	C10—H10	0.9500
			
C4—O1—C5	117.41 (9)	C10—C5—C6	122.42 (11)
C4—N1—C1	114.73 (11)	C10—C5—O1	118.01 (10)
C4—N2—C3	114.50 (11)	C6—C5—O1	119.37 (11)
O2—N3—O3	123.73 (11)	C5—C6—C7	118.97 (11)
O2—N3—C9	118.44 (11)	C5—C6—H6	120.5
O3—N3—C9	117.82 (11)	C7—C6—H6	120.5
N1—C1—C2	122.68 (13)	C6—C7—C8	120.65 (11)
N1—C1—H1	118.7	C6—C7—H7	119.7
C2—C1—H1	118.7	C8—C7—H7	119.7
C1—C2—C3	116.25 (12)	C9—C8—C7	117.91 (11)
C1—C2—H2	121.9	C9—C8—H8	121.0
C3—C2—H2	121.9	C7—C8—H8	121.0
N2—C3—C2	122.96 (12)	C8—C9—C10	123.55 (11)
N2—C3—H3	118.5	C8—C9—N3	118.55 (11)
C2—C3—H3	118.5	C10—C9—N3	117.88 (11)
N1—C4—N2	128.87 (12)	C5—C10—C9	116.49 (11)
N1—C4—O1	118.10 (11)	C5—C10—H10	121.8
N2—C4—O1	113.03 (10)	C9—C10—H10	121.8
			
C4—N1—C1—C2	−0.6 (2)	O1—C5—C6—C7	175.68 (10)
N1—C1—C2—C3	0.5 (2)	C5—C6—C7—C8	−0.65 (18)
C4—N2—C3—C2	−0.3 (2)	C6—C7—C8—C9	−0.02 (18)
C1—C2—C3—N2	0.0 (2)	C7—C8—C9—C10	0.66 (18)
C1—N1—C4—N2	0.2 (2)	C7—C8—C9—N3	−178.15 (11)
C1—N1—C4—O1	179.32 (11)	O2—N3—C9—C8	171.09 (11)
C3—N2—C4—N1	0.3 (2)	O3—N3—C9—C8	−7.71 (18)
C3—N2—C4—O1	−178.92 (11)	O2—N3—C9—C10	−7.78 (17)
C5—O1—C4—N1	−0.16 (16)	O3—N3—C9—C10	173.41 (13)
C5—O1—C4—N2	179.13 (10)	C6—C5—C10—C9	−0.15 (17)
C4—O1—C5—C10	−94.96 (13)	O1—C5—C10—C9	−175.15 (10)
C4—O1—C5—C6	89.88 (13)	C8—C9—C10—C5	−0.57 (18)
C10—C5—C6—C7	0.74 (18)	N3—C9—C10—C5	178.25 (10)
